# Evaluation of Deltamethrin in Combination of Piperonyl Butoxide (PBO) against Pyrethroid Resistant, Malaria Vector, *Anopheles stephensi* in IRS Implementation: an Experimental Semi-Filed Trial in Iran

**Published:** 2017-12-30

**Authors:** Fatemeh Nikpour, Hassan Vatandoost, Ahmad Ali Hanafi-Bojd, Ahmad Raeisi, Mansour Ranjbar, Ahmad Ali Enayati, Mohammad Reza Abai, Mansoreh Shayeghi, Abdol Rasoul Mojahedi, Abolghasem Pourreza

**Affiliations:** 1 Department of Medical Entomology and Vector Control, School of Public Health, Tehran University of Medical Sciences, Tehran, Iran; 2 Department of Environmental Chemical Pollutants, Institute for Environmental Research, Tehran University of Medical Sciences, Tehran, Iran; 3 Malaria Control Department, Ministry of Health and Medical Education, Tehran, Iran; 4 Independent Malaria Consultant, Tehran, Iran; 5 Department of Medical Entomology and Vector Control, School of Public Health, Mazandaran University of Medical Sciences, Sari, Iran; 6 Provincial Health Center, Bandar Abbas University of Medical Sciences, Bandar Abbas, Iran

**Keywords:** Insecticide resistance, *Anopheles stephensi*, Deltamethrin, Piperonyl butoxide, IRS

## Abstract

**Background:**

The aim of this study was to evaluate different concentrations of deltamethrin combined with formulated piperonyl butoxide (PBO) synergist on various surfaces against the wild strain of *Anopheles stephensi*, the main malaria vector in Southern Iran under semi-field condition.

**Methods:**

Four concentrations of deltamethrin WG 25% (Tagros) and PBO 800EC-UV (Endura) were prepared and sprayed on the pre-designed surfaces in accordance with WHO alliance line of the IRS Micronair®. The WHO’s recommended bioassay kit and method was used during this study.

**Results:**

Comparing the mortality rate of mosquitoes, the results showed a significant difference between months after treatment of IRS (Indoor Residual Spraying) (P< 0.05) but didn’t show any significant differences between days during the first and second months (P> 0.05).

Statistical test revealed a significance difference between mortality rate of mosquitoes in exposing to concentrations of 1 and 4 (P< 0.05) which demonstrated effect of synergizing PBO on mortality rate.

**Conclusion:**

This research as the first semi-field trial on deltamethrin added to different concentrations of formulated PBO for IRS, indicates that deltamethrin+10X PBO is more effective than other concentrations. Therefore, using synergists can be suggested as a new tool for prevention of pyrethriod resistance, although more studies are recommended.

## Introduction

Noticeable reduction (90%) in incidence and mortality of global malaria became one of aims of 2030 (1). Instead, the development of resistance to insecticides is probably the greatest threat to defeat malaria vectors control program. Pyrethroids are the main chemical components used in malaria vector control programs. The best methods for using pyrethroids are long lasting insecticidal nets (LLIN) and indoor residual spraying (IRS) (2). But increasing use and coverage of IRS and LLIN are causing more resistant mosquitoes which can finally undermine the success of these methods (3).

Vector Control Advisory Group (VCAG) has been established by WHO for advising new tools and approaches to vector control (4). The national malaria control program of the Islamic Republic of Iran was focused on controlling malaria however, in 2006 elimination became the focus along with the National Strategic Plan on elimination of local transmission (5). The action towards reducing malaria resulted in identification of only 167 indigenous (local) cases in 2015, that were almost all found in the three south-eastern provinces i.e Sistan and Baluchestan, Hormozgan and Kerman (6). In these areas, *Anopheles culicifacies* Giles s.l., *Anopheles dthali* Patton, *Anopheles fluviatilis* James s.l., *Anopheles stephensi* Liston and *Anopheles superpictus* Grassi are known to be proven malaria vectors, while there is also report of sporozoite infection of *Anopheles pulcherrimus* Theobald (7–9).

Several researches on insecticide resistance monitoring, revealed resistance status of *Anopheles* mosquitoes to a wide range of insecticides in Iran. *An. stephensi* resistance to insecticides (DDT, dieldrin and malathion) was first reported in 1957, 1960 and 1976 respectively. The results of susceptibility tests of the most recent report of pyrethroid resistance of this species in Iran (10) indicated that *An. culicifacies* is tolerant/resistant to DDT, dieldrin, propoxur (11), malathion (12).

*Anopheles dthali* has been known as resistant to DDT and dieldrin in Iran, but current studies show that *An. dthali* is susceptible to all tested insecticides from organochlorine, organophosphate, carbamates and pyrethroids (13). The tolerance to deltamethrin in this species (9, 14) is also a noticeable. Resistance to pyrethroids in *Anopheles* mosquitoes appears to be effected by target site insensitivity knock down resistance (kdr) and metabolic mechanism caused by mixed-function oxidases (MFO) (15–16).

Insects, in general, despite their susceptibility to insecticides, contain enzymes for metabolizing xenobiotic compounds and converting them to a non-toxic one that are finally removed through excretion. Degradation or metabolism of insecticides are inhibited by PBO through blocking action, making it more effective. A great advantage of adding PBO to LLIN is the increased activity of pyrethoids in susceptible insects. PBO also increases the activity of pyrethroids in susceptible insects, so the addition of PBO to LLIN has an advantage, even in areas where there is no resistance. Some studies have shown the impact of PBO resistances to pyrethroids in malaria vectors (17–18). Also, there were some laboratory and field trials in which PBO added to LLIN or larvicide component. The results of the latter showed PBO suppressed resistance to pyrethroid insecticides in different populations of Culicidae, indicating that oxidases and/or esterases play an important role in the reduction of pyrethroids toxicity (19–20).

Despite laboratory and field evaluation of PBO efficiency in LLINs and larvicides, so far there has not been any study on using this combination in IRS. Therefore, this study was aimed to evaluate insecticidal activity of different concentrations of deltamethrin combined with formulated PBO synergistic on various surfaces against the wild strain of *An. stephensi*, the malaria vector in southern Iran under the semi-field condition.

## Materials and Methods

### Preparation of the artificial surfaces

**Fig. 1.**
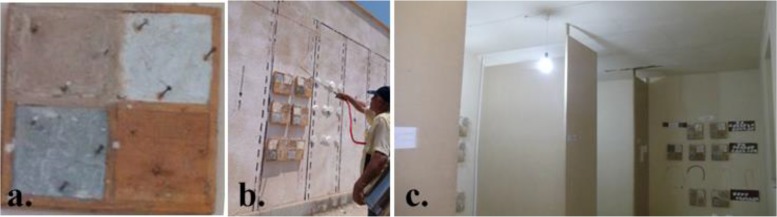
a. Wooden container with four different surfaces, b. Spraying operation, c. Install contair after spraying in room which seprated into four parts

Initially, 24 wooden containers with dimensions of 5×40×40cm, were divided into four parts and each part had three spikes for holding cones used for bioassay test (Fig. 1a). Cement, plaster, clay and wood surfaces were placed in each the wooden container and left to dry at room temperature. These containers were treated with insecticide and different concentrations of deltamethrin+synergist, while four untreated control containers were maintained (21).

### Concentrations

Four concentrations of deltamethrin WG 25% (Tagros) and PBO 800EC-UV (Endura) were prepared as follows:

-Concentration 1 (Con 1) deltamethrin (without PBO)
-Concentration 2 (Con 2) deltamethrin: PBO= 1: 3
-Concentration 3 (Con 3) deltamethrin: PBO= 1: 5
-Concentration 4 (Con 4) deltamethrin: PBO= 1:10

### Residual spraying

Five replicates of the containers have been installed on the wall and treated by different concentrations according to WHO alliance line of the IRS Micronair®. Insecticide was sprayed using a compression sprayer recommended by WHO for the IRS which is equipped with a pressure gauge and HSS-8002 nozzles tips with regulator set at 24–55 PSI. Each concentration was dissolved in 10 liters of water in compression sprayer tanks. The sprayer discharge rate was set to 755 to 780ml/min. The spray duration was adjusted to spray 19m^2^ in one minute (21). The operation was done by an expert under supervision (Fig. 1b). The containers treated with different concentrations were then allowed to dry at room temperature and installed vertically on the wall in four separate rooms (Fig. 1c).

### Mosquito species tested

*Anopheles stephensi* larvae were collected from Hormoodar village (27°19′14.72″N, 56°19′14.80″E), in the south of Bandar Abbas city during August 2015–January 2016 and were transferred to the insectary of Bandar Abbas Research Station as WHO collaborating Center for Malaria Training. The larvae were reared into F1 generation for subsequent tests.

### Adult susceptibility tests

Insecticide susceptibility tests were carried out under laboratory conditions against *An. stephensi* with deltamethrin 0.05% (diagnostic dose) impregnated paper provided by WHO. The procedure of test was followed according to WHO (22).

### Bioassay tests

The bioassay tests were carried out for evaluation of residual effect of different concentrations using standard WHO cones. The cones were fitted on different treated surfaces using rubber band. About 10–12 sugar-fed, 3–5 days old female mosquitoes were gently released into each cone at the vertical position. The mosquitoes were exposed for 30mins to each treated surfaces in five different replicates. The same procedures were carried out for control container. At the end of exposure time, the adults were transferred into clean cups with cotton wool pad containing 10% sucrose solution and were kept in the insectary for 24h recovery period, the time for recording the mortality rate.

Contact bioassay tests were carried out on days 1, 5, 15, 30, 45, 60, 105 and 120 after treatment. Relative humidity and temperature of the test rooms were recorded during the bioassay experiments (23).

### Statistical analysis

Data obtained from different replicates were collected for each surface. The mortality rate under 80% was considered as threshold level (24). The mortality rate rates were transformed into the Arc Sin √P. ANOVA test was used for comparison. Tests with control mortality rate between 5 and 20%, were corrected using Abbott’s formula (25).

## Results

The susceptibility tests of *An. stephensi* against diagnostic dose of deltamethrin (0.05%) resulted in 91% mortality rate. It means this species is a candidate of resistance to this insecticide according to the new WHO criteria.

Results of bioassay test on different surfaces during 120 days were as follows:

### Mortality rate in plaster surface

Results of bioassay test on plaster showed 80–100% mortality rate of *An. stephensi* during the first month of treatment for all concentrations. This ratio reduced to 36% in concentration Con 1 and Con 3 after 120 days of treatment. These results indicated that deltamethrin had a residual effect of about 2.5 months on Con 4 while the others had a residual effect around 1–1.5 months (Table 1). There was no significant difference for plaster surface between different concentrations after 120 days of treatment (P> 0.05).

**Table 1.** Persistence of deltamethrin with/without piperonyl but oxide on plaster surface against *Anopheles stephensi*, 2015–2016

**Days after spraying**	**Concentrations**												**Control**		

	**Concentration 1**			**Concentration 2**			**Concentration 3**			**Concentration 4**					

	**Total**	**Dead**	**Mortality ±SE**	**Total**	**Dead**	**Mortality ±SE**	**Total**	**Dead**	**Mortality ±SE**	**Total**	**Dead**	**Mortality ±SE**	**Total**	**Dead**	**Mortality**
**1**	52	47	90.4 ± 3.2	50	40	80.0 ± 0.6	50	43	86.0 ± 2.2	50	50	100.0 ± 0.0	40	3	7.5
**5**	50	50	100.0 ± 0.0	50	42	84.0 ± 2.8	50	50	100.0 ± 0.0	50	50	100.0 ± 0.0	40	5	12.5
**15**	51	51	100.0 ± 0.0	50	48	96.0 ± 4.0	50	48	96.0 ± 4.0	50	48	96.0 ± 2.6	40	2	5
**30**	50	46	92.0 ± 2.1	50	45	90.0 ± 2.7	50	45	90.0 ± 2.7	50	47	94.0 ± 2.4	40	2	5
**45**	51	41	80.4 ± 0.9	50	36	72.0 ± 3.0	50	36	72.0 ± 3.0	50	42	84.0 ± 3.9	40	3	7.5
**60**	50	37	74.0 ± 3.1	50	38	76.0 ± 5.1	50	38	76.0 ± 5.1	50	45	90.0 ± 2.9	40	2	5
**75**	50	32	64.0 ± 2.7	50	38	76.0 ± 2.0	50	38	76.0 ± 2.0	50	42	84.0 ± 2.2	40	4	10
**90**	50	27	54.0 ± 3.5	50	34	68.0 ± 2.2	50	29	58.0 ± 3.7	50	35	70.0 ± 3.3	40	0	0
**105**	50	25	50.0 ± 3.0	50	27	54.0 ± 1.9	50	27	54.0 ± 1.9	50	33	66.0 ± 2.6	40	1	2.5
**120**	50	18	36.0 ± 3.7	50	18	36.0 ± 1.9	50	18	36.0 ± 1.9	50	25	50.0 ± 1.6	40	2	5

### Mortality rate in thatch surface

Results of bioassay test on thatch showed 86–100% mortality rate of *An. stephensi* during the first month of treatment. Mortality rate after 120 days of treatment reduced to 45.1%, 50%, 50% and 47.5% in con.1, 2, 3 and 4 respectively. Mortality rate indicated that deltamethrin had a residual effect about two months on all concentrations except Con 1 (1.5 month) (Table 2).

**Table 2.** Persistence of deltamethrin with/without piperonyl but oxide on thatch surface against *Anopheles stephensi*, 2015–2016

**Days after spraying**	**Concentrations**												**Control**		

	**Concentration 1**			**Concentration 2**			**Concentration 3**			**Concentration 4**					

	**Total**	**Dead**	**Mortality ±SE**	**Total**	**Dead**	**Mortality ±SE**	**Total**	**Dead**	**Mortality ±SE**	**Total**	**Dead**	**Mortality ±SE**	**Total**	**Dead**	**Mortality**
**1**	50	50	100.0 ± 0.0	51	51	100.0 ± 0.0	49	48	98.0 ± 2.0	51	46	90.2 ± 3.6	40	3	7.5
**5**	51	50	98.0 ± 2.0	50	45	90.0 ± 3.2	50	50	100.0 ± 0.0	51	51	100.0 ± 0.0	40	5	12.5
**15**	50	50	100.0 ± 0.0	50	47	94.0 ± 2.5	50	47	94.0 ± 2.5	50	49	98.0 ± 2.0	40	2	5
**30**	50	43	86.0 ± 1.6	49	46	93.9 ± 4.0	49	46	93.9 ± 4.0	49	43	87.8 ± 3.7	40	2	5
**45**	51	42	82.4 ± 3.6	50	40	80.0 ± 3.2	50	40	80.0 ± 3.2	50	38	76.0 ± 2.0	40	3	7.5
**60**	50	38	76.0 ± 1.3	50	41	82.0 ± 1.9	50	41	82.0 ± 1.9	51	41	80.4 ± 4.6	40	2	5
**75**	50	33	66.0 ± 5.6	50	38	76.0 ± 4.2	50	38	76.0 ± 4.2	50	36	72.0 ± 6.7	40	4	10
**90**	50	21	42.0 ± 2.3	50	37	74.0 ± 3.0	50	32	64.0 ± 2.8	50	33	66.0 ± 9.4	40	0	0
**105**	52	23	44.2 ± 2.6	50	28	56.0 ± 1.9	50	28	56.0 ± 1.9	50	28	56.0 ± 4.2	40	1	2.5
**120**	51	23	45.1 ± 7.5	50	25	50.0 ± 2.8	50	25	50.0 ± 2.8	40	19	47.5 ± 3.3	40	2	5

### Mortality rate in cement surface

Results of bioassay test on cement showed 84–100% mortality rate of *An. stephensi* during the first month of treatment. Mortality rate after 120 days of treatment reduced to 50% in all concentrations. Mortality rate indicated that deltamethrin had a residual effect about 2.5 months on all concentrations except Con 1 (one month) (Table 3).

**Table 3.** Persistency of deltamethrin with/without piperonyl but oxide on cement surface against *Anopheles stephensi*, 2015–2016

**Days after spraying**	**Concentrations**												**Control**		

	**Concentration 1**			**Concentration 2**			**Concentration 3**			**Concentration 4**					

	**Total**	**Dead**	**Mortality ±SE**	**Total**	**Dead**	**Mortality ±SE**	**Total**	**Dead**	**Mortality ±SE**	**Total**	**Dead**	**Mortality ±SE**	**Total**	**Dead**	**Mortality**
**1**	50	42	84.0 ± 1.7	49	49	100.0 ± 0.0	50	50	100.0 ± 0.0	50	50	100.0 ± 0.0	40	3	7.5
**5**	51	45	88.2 ± 1.7	50	44	88.0 ± 2.0	50	50	100.0 ± 0.0	50	50	100.0 ± 0.0	40	5	12.5
**15**	50	50	100.0 ± 0.0	50	50	100.0 ± 0.0	50	50	100.0 ± 0.0	50	50	100.0 ± 0.0	40	2	5
**30**	50	46	92.0 ± 2.0	49	45	91.8 ± 3.5	49	45	91.8 ± 3.5	50	45	90.0 ± 3.2	40	2	5
**45**	50	37	74.0 ± 2.2	50	42	84.0 ± 2.2	50	42	84.0 ± 2.2	50	47	94.0 ± 2.4	40	3	7.5
**60**	49	36	73.5 ± 1.8	50	40	80.0 ± 3.2	50	40	80.0 ± 3.2	50	42	84.0 ± 2.2	40	2	5
**75**	51	36	70.6 ± 5.8	50	42	84.0 ± 2.6	50	42	84.0 ± 2.6	50	42	84.0 ± 2.2	40	4	10
**90**	49	34	69.4 ± 3.1	50	35	70.0 ± 3.3	50	32	64.0 ± 2.4	50	37	74.0 ± 2.7	40	0	0
**105**	50	32	64.0 ± 2.8	50	27	54.0 ± 1.6	50	27	54.0 ± 1.6	50	35	70.0 ± 3.5	40	1	2.5
**120**	50	25	50.0 ± 1.8	50	25	50.0 ± 3.2	50	25	50.0 ± 3.2	50	27	54.0 ± 2.7	40	2	5

### Mortality rate in wood surface

Results of bioassay test on wood showed 84–100% mortality rate of *An. stephensi* during the first month of treatment. Mortality rate after 120 days of treatment reduced to 46% in Con 2 and Con 3. Mortality rate indicated that deltamethrin had a residual effect about two months using Con 4, 1.5 month for Con 2 and Con 3, while one month using Con 1 (Table 4).

**Table 4.** Persistence of deltamethrin with/without piperonyl but oxide on wood surface against *Anopheles stephensi*, 2015–2016

**Days after spraying**	**Concentrations**												**Control**		

	**Concentration 1**			**Concentration 2**			**Concentration 3**			**Concentration 4**					

	**Total**	**Dead**	**Mortality ±SE**	**Total**	**Dead**	**Mortality ±SE**	**Total**	**Dead**	**Mortality ±SE**	**Total**	**Dead**	**Mortality ±SE**	**Total**	**Dead**	**Mortality**
**1**	49	46	93.9 ± 2.5	50	50	100.0 ± 0.0	51	51	100.0 ± 0.0	51	51	100.0 ± 0.0	40	3	7.5
**5**	50	42	84.0 ± 2.2	50	45	90.0 ± 0.0	50	50	100.0 ± 0.0	50	50	100.0 ± 0.0	40	5	12.5
**15**	51	51	100.0 ± 0.0	50	47	94.0 ± 2.5	50	47	94.0 ± 2.5	50	48	96.0 ± 2.3	40	2	5
**30**	49	49	100.0 ± 0.0	49	49	100.0 ± 0.0	49	49	100.0 ± 0.0	49	47	95.9 ± 2.4	40	2	5
**45**	51	39	76.5 ± 2.2	50	40	80.0 ± 3.2	50	40	80.0 ± 3.2	51	45	88.2 ± 5.8	40	3	7.5
**60**	49	34	69.4 ± 1.8	50	38	76.0 ± 2.5	50	38	76.0 ± 2.5	50	42	84.0 ± 2.4	40	2	5
**75**	50	35	70.0 ± 2.4	50	38	76.0 ± 4.2	50	38	76.0 ± 4.2	50	38	76.0 ± 4.2	40	4	10
**90**	50	31	62.0 ± 4.7	50	34	68.0 ± 2.0	50	32	64.0 ± 3.3	50	33	66.0 ± 2.4	40	0	0
**105**	50	23	46.0 ± 4.1	50	30	60.0 ± 1.3	50	30	60.0 ± 1.3	50	28	56.0 ± 3.6	40	1	2.5
**120**	50	25	50.0 ± 2.3	50	23	46.0 ± 3.0	50	23	46.0 ± 3.0	50	26	52.0 ± 1.2	40	2	5

Results of bioassay test using different concentrations during 120 days were as follows:

### Mortality rate of Concentration 1

**Fig. 2.**
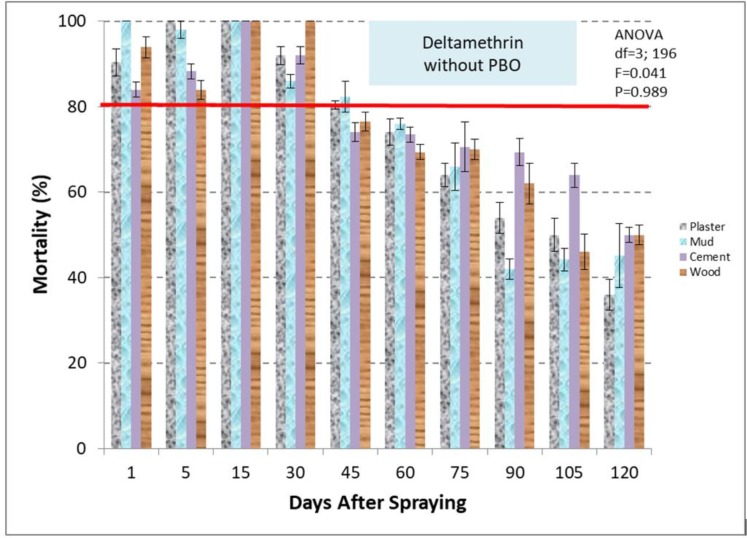
Comparison of deltamethrin persistence without piperonyl but oxide on different surfaces against *Anopheles stephensi*, 2015–2016

Mortality rate of *An. stephensi* on different surfaces ranged from 84–100% during the first month of treatment. This value has dropped to 50% on cement and wood surfaces after 120 days of spraying. However, on plaster and thatch surfaces, mortality rate was reduced to less than 50% after 90 and 105 days after treatment. So deltamethrin had a residual effect of about 1.5 month on thatch and plaster, one month on other surfaces (Fig. 2).

### Mortality rate of Concentration 2

**Fig. 3.**
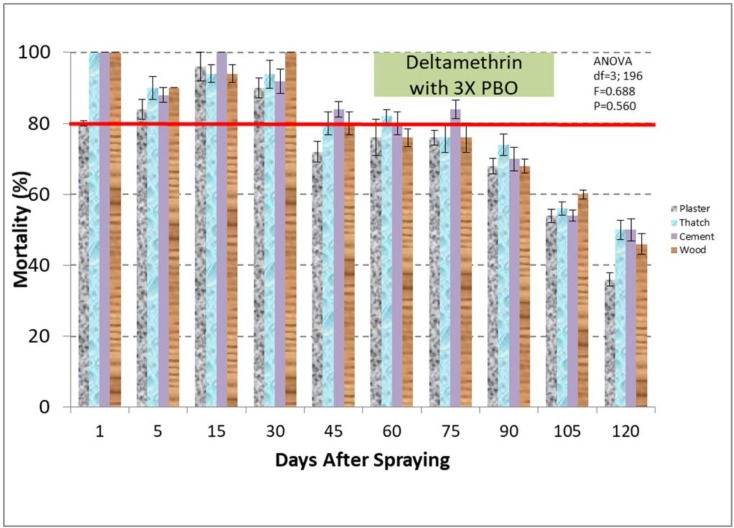
Comparison of deltamethrin persistence with 3X piperonyl but oxide and deltamethrin on different surfaces against *Anopheles stephensi*, 2015–2016

Mortality rate of *An. stephensi* on different surfaces ranged from 80–100% during the first month of treatment. Mortality rate after 120 days of treatment was 50% on thatch and cement surfaces but mortality rate on plaster and wood was less than 50% after 105 after treatment so based on indicating that deltamethrin has a residual effect of about 2.5 months on cement, two months on thatch, 1.5 month on wood and one month on plaster surface (Fig. 3).

### Mortality rate of Concentration 3

**Fig. 4.**
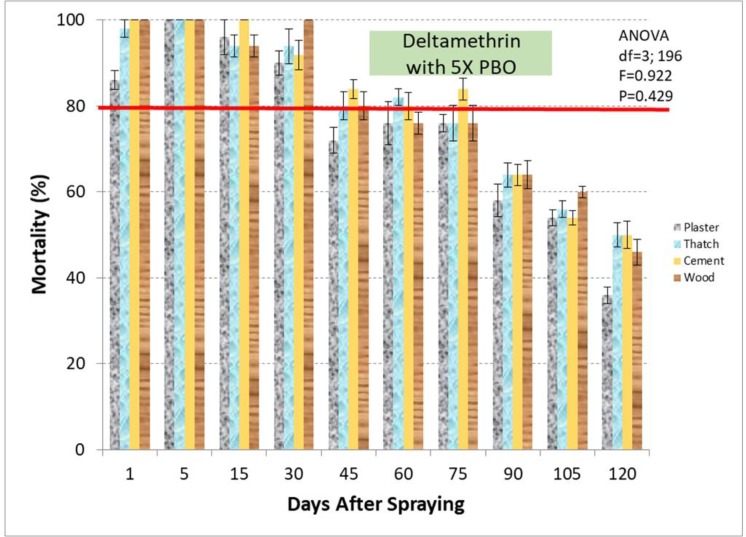
Comparison of deltamethrin persistence with 5X piperonyl but oxide on different surfaces against *Anopheles stephensi*, 2015–2016

Mortality rate of *An. stephensi* on different surfaces ranged from 86–100% during the first month of treatment. Mortality rate after 120 days of treatment was 50% on thatch and cement surfaces but mortality rate on plaster and wood surfaces were less than 50% after day 105 of treatment. The results showed that deltamethrin has a residual effect of about 2.5 months on cement, two months on thatch, 1.5 month on wood and one month on plaster surface (Fig. 4).

### Mortality rate of Concentration 4

**Fig. 5.**
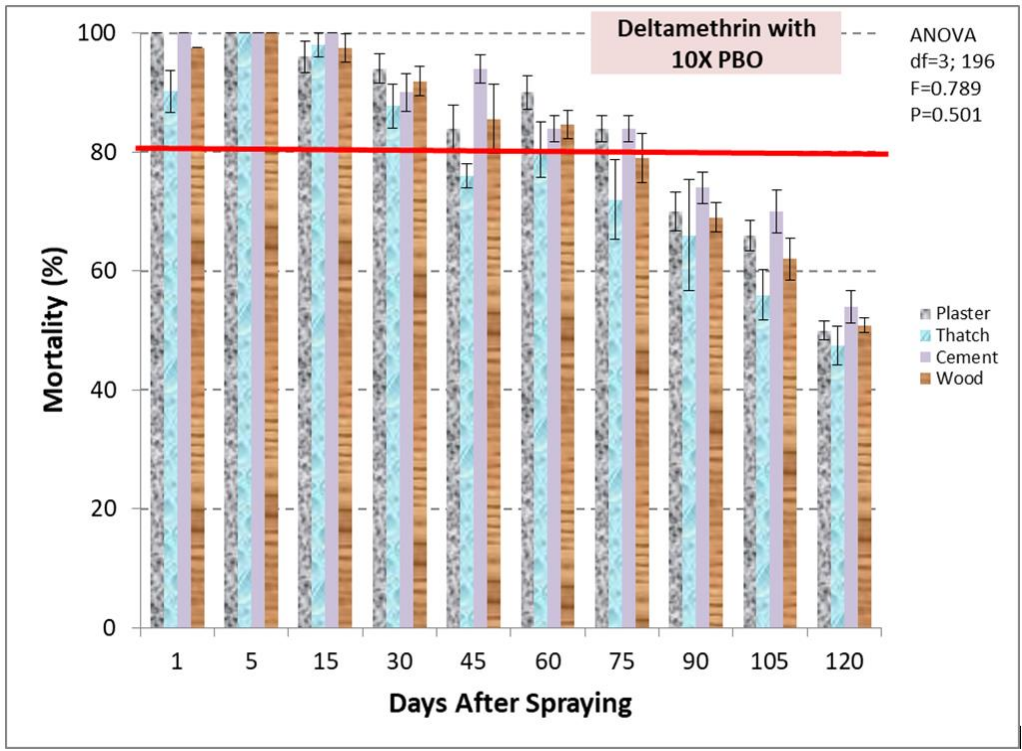
Comparison of deltamethrin persistence with 10X piperonyl but oxide on different surfaces against *Anopheles stephensi*, 2015–2016

Mortality rate of *An. stephensi* on different surfaces ranged from 88–100% during the first month of treatment. Mortality rate after 120 days of treatment was equal to or more than 50% on plaster, cement and wood surfaces but mortality rate on thatch was less than 50% after day 105 of treatment. Based on these results deltamethrin has a residual effect of about 2.5 months on plaster and cement and two months on thatch and wood surfaces (Fig. 5).

Comparing the mortality rate of mosquitoes, there was a significant difference between months after treatment (P< 0.05) but there were no significant differences between days in the first and second months (P> 0.05).

Statistical test revealed a significant difference in mortality rate of mosquitoes in exposure to Con 1 and others (P< 0.0001), but no significant difference was found between Con 2 and Con 3 (P> 0.05).

Tukey’s test showed that there was a significant difference between mortality rate of mosquitoes on cement and other surfaces (P< 0.05), while there was no significant between other three surfaces (P> 0.05).

## Discussion

At present, pyrethroids are being used for IRS and in mosquito nets and various products worldwide (3). There is no alternative insecticide for the treatment of nets other than pyrethroids synergistic nets. Among products being evaluated by the Pesticide Evaluation Scheme (WHOPES) were mosquito nets containing a pyrethroid and a compound of an unrelated class (e.g. chlorfenapyr or pyriproxyfen) and clothianidin IRS formulated with or without pyrethroid (27–28).

Deltamethrin+PBO for use in IRS, has been a proposed product in order to overcome the appearance of resistance. This combined product could be used as a vector control tool in country programs. This tool is highly recommended in countries that are in elimination phase or have reported insecticide resistance due to vector control strategy being a key strategy.

The results of this study showed that although mortality rates in different concentrations of deltamethrin+PBO decreased during 120 days after treatment, mortality rate in day 120 in all of them was higher than deltamethrin without PBO (Figs. 2–5). Regardless of surface type, there was also an eligible difference in mortality rate between deltamethrin without PBO (Con 1) and deltamethrin+ PBO= 1:10 (Con 4) concentration against *An. stephensi* field strain (P< 0.0001). Therefore, it can be concluded that PBO had a positive effect on the efficacy of insecticide.

Exito-repellency effect of deltamethrin may be the reason for different mortality rates (90–100%) between different surfaces and concentrations in the first month of the study.

The study on plaster surfaces showed a significant difference between mortality rate on Con 1 and Con 4 on both days 1 and 120 after treatment, but this difference was only significant in day 1 on cement, thatch and wood surfaces. This results is in line with the study in Benin that evaluated PermaNet 3.0 (deltamethrin+PBO) against pyrethroid-resistant *An. gambiae* and *Cx. quinquefasciatus* in an experimental hut. They found a negligible difference between the mortality rate of PermaNet 2.0 (deltamethrin) and PermaNet 3.0 before and after the 20 times washing (29).

In some researches in African countries which was proved kdr and metabolic resistance, tricomponent of LLIN were used. These nets include pyrethroid+PBO and other group of insecticides with different mechanisms of action such as pyrole chlorfenapyr or neon-icotinoid. They found tricomponents had more insecticidal activity than one component LLIN on pyrethroid-resistant *An. gambiae*, *Ae. aegypti*, *An. funestus* and *Cx. quinquefasciatus* (30–33).

A study conducted on a pyrethroid resistant strain of *Cx. pipiens* (3.8 to 38.4 folds) evaluated mixture of pyrethriod larvicides and PBO (20). They found PBO suppressed resistance to pyrethroid insecticides (>90%) in field populations indicating that oxidases and/or esterases play an important role in the reduction of pyrethroids toxicity. Another survey conducted to assay larviciding impact of a mixture of stock solution of PBO and deltamethrin in 6:1 ratio on resistant strains (4–21 folds) of *Ae. aegypti*, *An. culicifacies*, *An. stephensi*, *An. vagus*, *Cx. tritaeniorhynchus*, *Cx. pipiens*, revealed that PBO suppressed resistance between 75–95% (26). It can be concluded that lower mortality rate indicates resistance which can result in better efficacy of PBO. Although, in this study, we had 91% mortality rate in the tested strain which was not resistance strain but significant differences were found in mortality rates between some concentrations (Figs. 2–5). Both above mentioned studies used technical PBO under laboratory condition, but we applied a formulated product under semi-field condition. These differences may also affect the results.

Several study results revealed that mortality rate in non-sorbent (wood) and sorbent surfaces (mud, thatch, plaster and cement) had no significant differences in mortality rate.

The absorption rate on different surfaces (wood, plaster, mud and cement) had notable variability in mortality rate on parous surfaces, as have been reported in other studies (21, 34) but the result of this study revealed differences in absorption rate on parous surfaces in different concentrations (Con 1 and Con 3 and Con 4) (Tables 1–4) which can be the effect of PBO. It seems that the moderate and high concentration of PBO (Con 3 and Con 4) had effected the high level of mortality rate on the first day of treatment however in Con 1, the high level of mortality started on day 15 of treatment. These results indicated that the presence of synergist has led to a decrease in absorption therefore resulting in high mortality rate from the beginning of treatment in comparison with absence of synergist (Con 1).

## Conclusion

In conclusion, considering that the strain of *An. stephensi* used in this study was not resistance strain and the PBO could not result in significant difference in mortality rate after day 120, however the results suggest that the combination of deltamethrin+PBO can be more effective in mortality rate of resistant *An. stephensi.* Also PBO was observed to be more functional on porous substrates, while higher concentration of PBO seems to be more effective. However, more studies on the strains with higher resistant ratio can prove our results. This method can be considered as a new tool for malaria vector control, although more studies are recommended under field condition.
